# Common Genetic Variations in Patched1 (*PTCH1*) Gene and Risk of Hirschsprung Disease in the Han Chinese Population

**DOI:** 10.1371/journal.pone.0075407

**Published:** 2013-09-20

**Authors:** Yang Wang, Jun Wang, Weihua Pan, Ying Zhou, Yongtao Xiao, Kejun Zhou, Jie Wen, Tingxi Yu, Wei Cai

**Affiliations:** 1 Department of Pediatric Surgery, Xin Hua Hospital, School of Medicine, Shanghai Jiao Tong University, Shanghai, China; 2 Shanghai Key Laboratory of Pediatric Gastroenterology and Nutrition, Shanghai, China; 3 Shanghai Institute for Pediatric Research, Shanghai, China; Tongji Medical College, Huazhong University of Science and Technology, China

## Abstract

Hirschsprung disease (HSCR) is the most frequent genetic cause of congenital intestinal obstruction with an incidence of 1:5000 live births. In a pathway-based epistasis analysis of data generated by genome-wide association study on HSCR, specific genotype of Patched 1 (*PTCH1*) has been linked to an increased risk for HSCR. The aim of the present study is to examine the contribution of genetic variants in *PTCH1* to the susceptibility to HSCR in Han Chinese. Accordingly, we assessed 8 single nucleotide polymorphisms (SNPs) within *PTCH1* gene in 104 subjects with sporadic HSCR and 151 normal controls of Han Chinese origin by the Sequenom MassArray technology (iPLEX GOLD). Two of the eight genetic markers were found to be significantly associated with Hirschsprung disease (rs357565, allele *P* = 0.005; rs2236405, allele *P* = 0.002, genotype *P* = 0.003). Both the C allele of rs357565 and the A allele of rs2236405 served as risk factors for HSCR. During haplotype analysis, one seven-SNP-based haplotype was the most significant, giving a global *P* = 0.0036. Our results firstly suggest common variations of *PTCH1* may be involved in the altered risk for HSCR in the Han Chinese population, providing potential molecular markers for early diagnosis of Hirschsprung disease.

## Introduction

Hirschsprung disease (HSCR) affects approximately 1:5000 live births (the highest incidence is observed in Asian population, 2.8:10000 live births) and is characterized by the absence of parasympathetic ganglion cells (aganglionosis) in the hindgut, leading to tonic contraction of the affected segment, intestinal obstruction and massive distension of the proximal bowel [1,2]. HSCR is considered as a neurocristopathy since it is caused by the premature arrest of the craniocaudal migration of vagal neural crest cells in terminal regions of the gut between the 5^th^ and 12^th^ week of gestation to form the enteric nervous system (ENS) [1]. It can be anatomically categorized into three types, according to the extent of aganglionosis: short-segment HSCR (S-HSCR, 80% of cases) which affects the rectum and a short portion of the colon, long-segment HSCR (L-HSCR, 15% of cases) which affects longer tracts of the colon and total colonic aganglionosis (TCA, 5% of cases) [3].

The genetic complexity observed in HSCR can be attributed to non-Mendelian inheritance in nature, low sex-dependent penetrance, variability in the length of the ananglionic segment and involvement of multiple genetic and environmental factors [4,5]. Variants of at least eleven genes to date have been implicated in Hirschsprung disease including the two major ones, *RET* (receptor tyrosine kinase) and *EDNRB* (endothelin receptor type B), whereas these mutations account for only 50% of familial and up to 20% sporadic subjects with HSCR, and cumulatively explain a small proportion of the heritability [5]. Additionally, common variants in the regulatory region of *RET* were found to be involved in mediating susceptibility to HSCR by conferring an obvious reduction of the RET expression [6]. On the other hand, joint gene-gene effects (e.g. *RET* and *PHOX2B*; *RET* and *HOX* genes) may also have a substantial impact on the risk of Hirschsprung disease [7,8]. Recently, *NRG3* that performs important functions during neural development has been identified as a novel susceptibility gene in a whole exome sequencing study [9]. However, which of the candidate genes encompassed by these susceptibility loci mainly contribute to HSCR susceptibility remains unresolved.

Patched 1 (*PTCH1*) gene located on chromosome 9q22, 3 is expressed in three major isoforms (1, 1A and 1B), which are upregulated by transcription factor Gli [10]. Since *PTCH1* functions as a tumor suppressor, Danaee and colleagues have found that loss of *PTCH1* function due to mutation or deletion contributed to the genesis of basal cell carcinoma [11]. The encoded protein, PTCH1, serves as Hedgehog (Hh)-binding receptor, and is supposed to be the key regulator of the Hedgehog (Hh) signaling pathway that has been implicated in mediating proliferation and differentiation of the enteric neural crest cells (ENCCs) [12,13]. Reduced PTCH1 causes overstimulation of Hh which can result in the hyperproliferation of epithelial cells [12]. By applying canonical correlation analysis, Ngan et al. [13] found mutations within *PTCH1* conferred higher risk to Hirschsprung disease, and deletion of *Ptch1* in mouse ENCCs led to premature gliogenesis and reduction of ENCC progenitors in mutant bowels. In the present study, we aimed to evaluate whether common variants in the *PTCH1* gene might contribute to the altered risk of Hirschsprung disease in the Han Chinese population.

## Materials and Methods

### Ethics Statement

This study was approved by the institution review board of Xinhua Hospital, School of Medicine, Shanghai Jiao Tong University. We initiated this research in accordance with the defined protocols in which the design and performance of current study involving human subjects were clearly described. Written informed consent was obtained from parents of all participants after the procedure had been fully explained. All data were recorded anonymously, but the data were destroyed if the participants asked to withdraw their file.

### Subjects

The characteristics of the study population are summarized in Table 1. We recruited 104 subjects with Hirschsprung disease (84 males and 20 females) and 151 normal controls (86 males and 65 females) in the present study. The mean age of HSCR cases was 1.14 ± 1.83 years, and the mean age of controls was 1.66 ± 1.05 years. All the subjects involved in the study were biologically unrelated Han Chinese in origin, and were enrolled from local Shanghai residents. All cases underwent resection between 2009 and 2012 at the Department of Pediatric Surgery, Xin Hua Hospital, Shanghai, China. The disease severity of each HSCR subject was categorized by the extent of aganglionosis. Short segment HSCR (S-HSCR) was defined as the aganglionic segment which did not extend beyond the upper sigmoid, and long segment HSCR (L-HSCR) was defined when aganglionosis extended proximal to the sigmoid region including total colonic aganglionosis (TCA) [1]. Out of the 104 HSCR cases, 86 cases (82.7%) were diagnosed as S-HSCR, 15 cases (14.4%) were L-HSCR and 3 cases (2.9%) were TCA. Diagnosis was confirmed by the histological examination of biopsy/surgical resection material for the absence of enteric nerve plexuses. Controls were randomly selected from the general population with no history of chronic constipation. Approval was received for the study from the ethics committee of Xin Hua Hospital and written informed consent was obtained from parents of all participants after the procedure had been fully explained. All DNA samples were extracted from peripheral blood using QIAamp DNA blood midi kit (Qiagen, Valencia, CA) according to the manufacturer’s protocol.

**Table 1:** Clinical characteristics of the subjects.

Characteristics	Case (n=104)	Control (n=151)
Male	84	86
Female	20	65
Age ± SD	1.14 ± 1.83	1.66 ± 1.05
S-HSCR, No. (%)	86 (82.7)	
L-HSCR, No. (%)	15 (14.4)	
TCA, No. (%)	3 (2.9)	

HSCR = Hirschsprung disease, L-HSCR = long-segment HSCR, S-HSCR = short-segment HSCR, TCA = total colonic aganglionosis.

### SNP selection and genotyping

**Figure 1:**
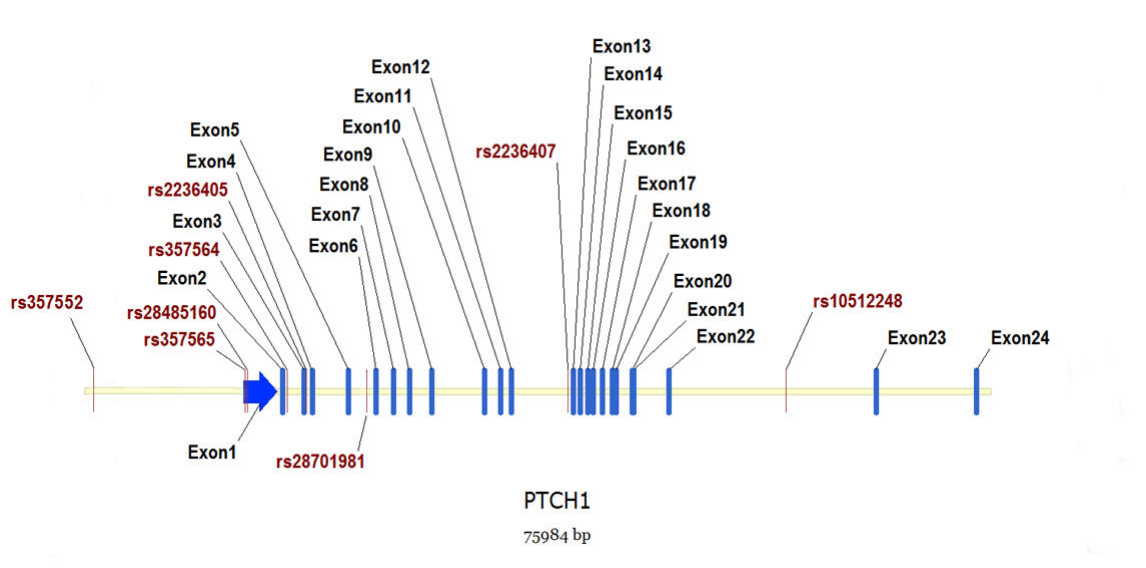
Distributions of the eight genetic polymorphisms in the genomic region of *PTCH1*. Red lines represent the SNPs genotyped; blue lines and arrow indicate all the 24 exons within *PTCH1*.

We recruited eight genetic polymorphisms, namely rs357552 and rs10512248, which had been reported by Ngan et al. [13], and six other SNPs (rs357565, rs28485160, rs357564, rs2236405, rs28701981 and rs2236407) from HapMap project database (http://www.hapmap.org) and dbSNP (http://www.ncbi.nlm.nih.gov/SNP/) to cover the region of *PTCH1*. The eight genetic markers involved in the study spanned ^~^ 73.4 kb region of *PTCH1*, with an average interval of ^~^ 9.2 kb: rs357565 and rs28485160 in the untranslated region of exon1, rs357564 in exon2, rs2236405 in exon3, and rs357552, rs28701981, rs2236407, and rs10512248 in the intronic region (Figure 1).

All genetic polymorphisms were genotyped using the MassARRAY iPLEX Gold technology (Sequenom, San Diego, CA). Briefly, PCR and single-base extension primers (SBE) were designed using the Assay Design Suite of Sequenom. The whole process was performed according to the manufacture’s instructions for the multiplex reaction, including the PCR amplification, the shrimp alkaline phosphatase (SAP) treatment, and the primer extension reactions using iPLEX Gold assay (Sequenom, San Diego, CA). Reaction products were then dispensed onto a 384-SpectroCHIP using the MassARRAY Nanodispenser and analyzed on the MassARRAY platform. Mass signals for the different alleles were captured by matrix-assisted laser desorption/ionization time-of-flight mass spectrometry (MALDI-TOF MS) with high accuracy. Typer Version 4.0 (Sequenom, San Diego, CA) was used to process raw data obtained from the assays.

### Genotyping quality control

We compared genotype call rates and concordance rates for each 384-well plate and the overall study, and examined contamination and reliability of the platform by including 30 sample duplicates and 4 blank wells (H_2_O) in each 384-well plate. The following criteria was used as a measure of acceptable genotyping: (1) call rate > 95% for each 384-well plate; (2) overall call rate by marker and by individual > 95%; (3) concordance rate for the duplicates ≥ 99.7%; (4) minor allele frequency (MAF) > 3% for each SNP; and (5) the call rate of the blank < 5% for each 384-well plate. The data for any SNP or individual failing these criteria were excluded from further analysis.

### Statistical analysis

SHEsis (http://analysis2.bio-x.cn/myAnalysis.php), a user-friendly platform particularly suited to association studies [14], was used to calculate Hardy-Weinberg equilibrium, allele and genotype frequencies. Odds ratio (OR) and 95% confidence interval (CI) were calculated on the website http://www.hutchon.net/ConfidOR.htm. We further recruited Haploview 4.1 [15] to estimate allelic distribution and linkage disequilibrium (LD), and included “*D*” as the standardized measure for all possible pairs of SNP loci. The program UNPHASED was used to estimate haplotype distribution [16]. All the *P* values in the present study were two-tailed and the significance level was set at *P* = 0.05. We corrected the *P* values of association analysis using a false discovery rate (FDR) controlling procedure [17] and employed Plink to execute the adjustment for age and gender factors in the genetic analysis [18]. Power calculations for our sample size were performed using the G*Power 3 program [19].

## Results

**Figure 2:**
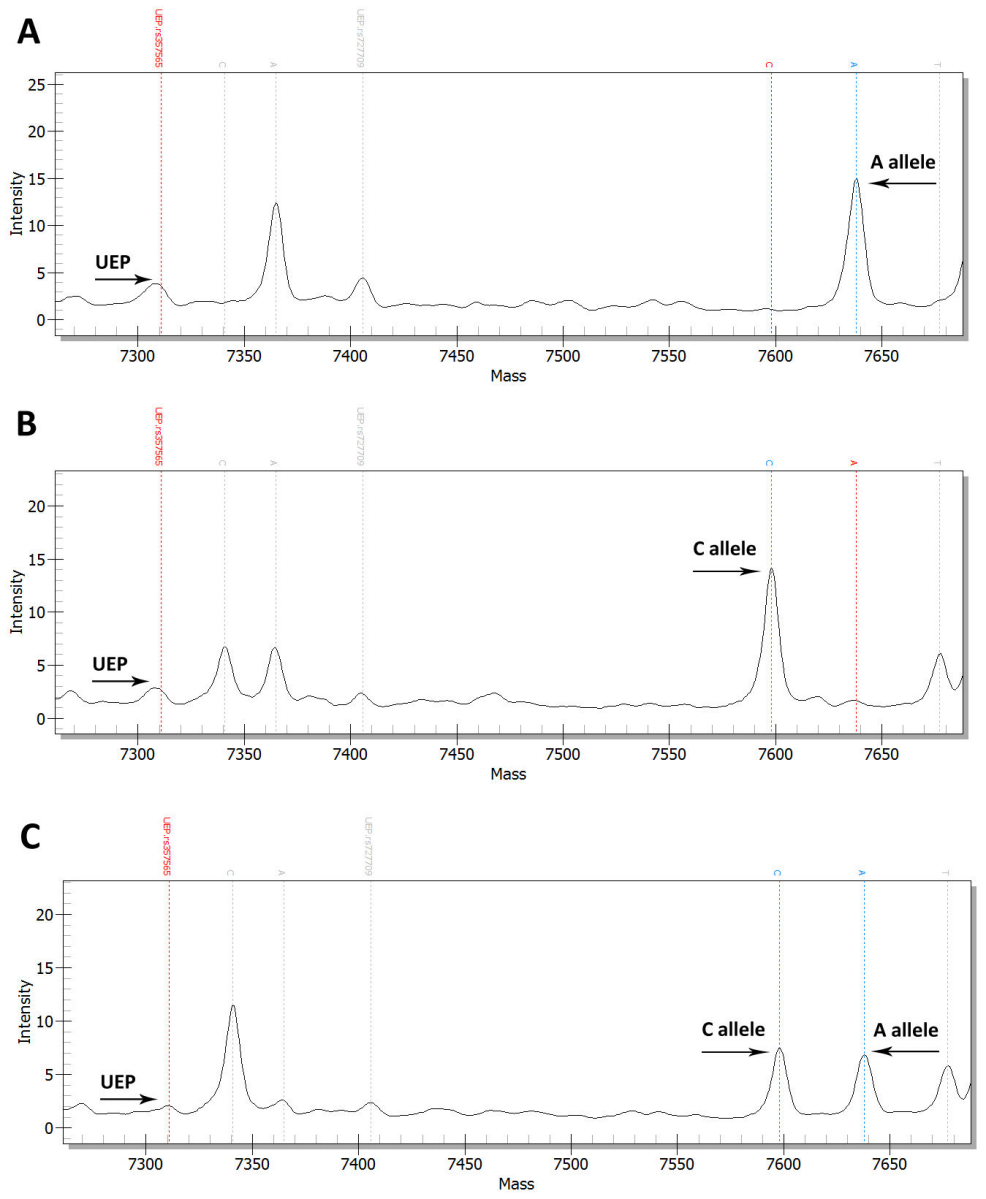
Representative mass spectra of the MassArray reactions including the three genotypes (A: A/A; B: C/C; C: A/C) of SNP rs357565. The alleles and UEP (unextended primer) are indicated with thick horizontal arrows.

As for the studied genetic markers, Hardy-Weinberg equilibrium tests were performed in cases and controls respectively. Genotype distributions were in Hardy-Weinberg equilibrium for all eight polymorphisms in either cases or controls (*P* > 0.05). For the 104 subjects with Hirschsprung disease and the 151 normal controls, we found that rs357565 and rs2236405 were significantly associated with Hirschsprung disease (rs357565, allele, *P* = 0.005, genotype, *P* = 0.015; rs2236405, allele, *P* = 0.002, genotype, *P* = 0.003). Representative mass spectra of the original MassArray reactions (e.g. SNP rs357565) are presented in Figure 2. Allele and genotype distributions of the 8 SNPs are shown in Table 2. The results observed in rs357565 and rs2236405 remained significant after the FDR correction (rs357565, allele, *P* = 0.018; rs2236405, allele, *P* = 0.019, genotype, *P* = 0.028). Of note, the C allele and CC genotype of rs357565 were more common in the case group compared to the control group (C allele, 87.3% versus 77.2%, OR = 0.49, 95% CI 0.30-0.81; CC genotype, 76.5% versus 59.1%), and likewise the A allele and AA genotype of rs2236405 showed significantly higher frequencies in the HSCR subjects than in the normal controls (A allele, 18.4% versus 9.0%, OR = 2.28, 95% CI 1.33-3.93; AA genotype, 6.1% versus 0%). Since there was a difference in the proportions of males and females between the two subject groups, PLINK software was included in the adjustment for age and gender factors, and the significance of the two markers remained after correction (*P* < 0.05).

**Table 2:** Allele and genotype distributions among HSCR patients and normal controls.

SNP ID	Genotype frequency(%)			H-W check *p* value*	*P* value*	FDR adjusted	Allele frequency(%)		X^2^	*P* value*	FDR adjusted	Odds Ratio (95%CI)
rs357552	AA	AG	GG				A	G				
Case	22 (21.8)	60 (59.4)	19 (18.8)	0.057	0.159	0.255	104 (51.5)	98 (48.5)	2.102	0.147	0.392	0.77 (0.54-1.10)
Control	49 (32.9)	75 (50.3)	25 (16.8)	0.682			173 (58.1)	125 (41.9)				
rs357565	AA	AC	CC				A	C				
Case	2 (2.0)	22 (21.6)	78 (76.5)	0.760	**0.015**	0.062	26 (12.7)	178 (87.3)	8.075	**0.005**	**0.018**	0.49 (0.30-0.81)
Control	7 (4.7)	54 (36.2)	88 (59.1)	0.724			68 (22.8)	230 (77.2)				
rs28485160	CC	CT	TT				C	T				
Case	2 (2.0)	39 (38.6)	60 (59.4)	0.126	0.469	0.536	43 (21.3)	159 (78.7)	0.605	0.437	0.582	1.20 (0.76-1.88)
Control	4 (2.8)	44 (31.2)	93 (66.0)	0.656			52 (18.4)	230 (81.6)				
rs357564	AA	AG	GG				A	G				
Case	30 (29.1)	50 (48.5)	23 (22.3)	0.803	0.585	0.585	110 (53.4)	96 (46.6)	1.095	0.295	0.591	1.21 (0.85-1.73)
Control	36 (24.2)	73 (49.0)	40 (26.8)	0.813			145 (48.7)	153 (51.3)				
rs2236405	AA	AT	TT				A	T				
Case	6 (6.1)	24 (24.5)	68 (69.4)	0.070	**0.003**	**0.028**	36 (18.4)	160 (81.6)	9.289	**0.002**	**0.019**	2.28 (1.33-3.93)
Control	0 (0.0)	26 (17.9)	119 (82.1)	0.236			26 (9.0)	264 (91.0)				
rs28701981	CC	CT	TT				C	T				
Case	15 (14.9)	49 (48.5)	37 (36.6)	0.851	0.369	0.492	79 (39.1)	123 (60.9)	0.624	0.429	0.687	1.16 (0.80-1.68)
Control	23 (15.8)	58 (39.7)	65 (44.5)	0.106			104 (35.6)	188 (64.4)				
rs2236407	AA	AG	GG				A	G				
Case	35 (35.0)	54 (54.0)	11 (11.0)	0.144	0.109	0.217	124 (62.0)	76 (38.0)	0.381	0.537	0.614	0.89 (0.61-1.29)
Control	65 (44.5)	59 (40.4)	22 (15.1)	0.165			189 (64.7)	103 (35.3)				
rs10512248	AA	AC	CC				A	C				
Case	39 (39.4)	53 (53.5)	7 (7.1)	0.052	0.072	0.191	131 (66.2)	67 (33.8)	0.049	0.825	0.825	0.96 (0.65-1.40)
Control	69 (47.3)	58 (39.7)	19 (13.0)	0.227			196 (67.1)	96 (32.9)				

* Pearson’s *p* value, FDR = false discovery rate, SNP = single nucleotide polymorphism, CI = confidence interval, HSCR = Hirschsprung disease.

The estimation of linkage disequilibrium for each pair of SNPs presented strong linkage disequilibrium (*D*’ > 0.7) for two groups of markers (rs357552-rs357565-rs28485160 and rs2236405-rs28701981-2236407-rs10512248) (Table 3) [20]. Moreover, haplotypes were omitted from analysis if the estimated haplotype probabilities were less than 3% in either the HSCR or normal control subjects. We therefore adopted the haplotype distributions for these SNPs in the later analysis.

**Table 3:** Estimation of linkage disequilibrium between the 8 SNPs.

	rs357552	rs357565	rs28485160	rs357564	rs2236405	rs28701981	rs2236407	rs10512248
rs357552		**0.96**	**0.92**	0.50	0.24	0.57	0.52	0.51
rs357565	0.18		**1.00**	0.42	0.02	0.30	0.31	0.22
rs28485160	0.17	0.06		0.58	0.21	**0.79**	**0.75**	0.69
rs357564	0.20	0.04	0.09		0.62	**0.73**	**0.71**	0.65
rs2236405	0.01	0.00	0.03	0.06		**0.90**	**0.87**	0.55
rs28701981	0.15	0.01	0.26	0.32	0.20		**0.96**	**0.90**
rs2236407	0.12	0.01	0.25	0.29	0.19	0.90		**0.94**
rs10512248	0.10	0.01	0.25	0.22	0.09	0.67	0.76	

For each pair of SNPs, *D*’ > 0.7 are shown in boldface, D' values are shown above and r^2^ values below the diagonal.

SNP = single nucleotide polymorphism.

We chose only those haplotypes with significant frequency discrepancies between HSCR and control groups (Table 4) for presentation. As the frequencies were higher in the HSCR group compared to the control group, several haplotypes were found to be correlated with an increased odds ratio for HSCR: the haplotype A-G (rs2236405-rs2236407, *P* = 0.006, OR 2.21, 95% CI 1.25-3.90), the haplotype A-C-C-A-C-G-C (rs357552-rs357565-rs28485160-rs2236405-rs28701981-rs2236407-rs10512248, *P* = 0.004, OR 4.66, 95% CI 1.48-14.64), and the haplotype A-C-C-G-A-C-G-C (rs357552-rs357565-rs28485160-rs357564-rs2236405-rs28701981-rs2236407-rs10512248, *P* = 0.002, OR 5.52, 95% CI 1.66-18.35). Moreover, the haplotype analysis of these genetic markers revealed some significant global *P* values (Table 5). One seven-SNP-based haplotype was the most significant, giving a global *P* = 0.0036.

**Table 4:** Estimated haplotype frequencies and association significance.

Haplotype*								Haplotype frequency (%)		X^2^	*p* value	Odds Ratio (95%CI)
rs357552	**rs357565**	rs28485160	rs357564	**rs2236405**	rs28701981	rs2236407	rs10512248	HSCR	Control			
A	A							24.39 (12.2)	68.00 (22.8)	8.705	**0.003**	0.47 (0.29-0.78)
	A	T						25.90 (13.1)	63.98 (23.0)	7.439	**0.006**	0.50 (0.31-0.83)
A	A	T						24.80 (12.8)	64.00 (23.0)	7.922	**0.005**	0.49 (0.29-0.81)
				A	C			32.21 (16.4)	26.00 (9.0)	6.614	**0.010**	2.04 (1.18-3.55)
				A		G		31.42 (16.2)	23.49 (8.1)	7.696	**0.006**	2.21 (1.25-3.90)
				A			C	23.28 (12.0)	18.63 (6.4)	4.569	**0.033**	1.99 (1.05-3.76)
				A	C	G		30.40 (15.7)	23.98 (8.3)	6.585	**0.010**	2.09 (1.18-3.69)
				A		G	C	24.13 (12.6)	18.00 (6.2)	5.563	**0.018**	2.13 (1.12-4.05)
				A	C	G	C	22.96 (12.0)	18.40 (6.3)	4.431	**0.035**	1.98 (1.04-3.77)
A	C	C		A	C	G	C	12.23 (6.6)	4.02 (1.5)	8.232	**0.004**	4.66 (1.48-14.64)
A	A	T		T	T	A	A	17.93 (9.6)	45.54 (16.5)	5.058	**0.025**	0.51 (0.29-0.92)
A	A	T	G	T	T	A	A	7.55 (4.1)	28.79 (10.4)	5.815	**0.016**	0.37 (0.16-0.85)
A	C	C	G	A	C	G	C	11.92 (6.4)	3.58 (1.3)	9.596	**0.002**	5.52 (1.66-18.35)

* Haplotypes were omitted from analysis if the estimated haplotype probabilities were less than 3%. CI = confidence interval, HSCR = Hirschsprung disease.

**Table 5:** Global p values of estimated haplotypes of the 8 SNPs within *PTCH1*.

Haplotype	Global *p* value*
rs357552-rs357565	**0.0126**
rs357565-rs28485160	**0.0240**
rs357552-rs357565-rs28485160	**0.0360**
rs2236405-rs28701981	**0.0361**
rs2236405-rs2236407	**0.0178**
rs2236405-rs10512248	**0.0387**
rs2236405-rs28701981-rs2236407	**0.0352**
rs357552-rs357565-rs28485160-rs2236405-rs28701981-rs2236407-rs10512248	**0.0036**
rs357552-rs357565-rs28485160-rs357564-rs2236405-rs28701981-rs2236407-rs10512248	**0.0263**

* Pearson’s *p* value, statistical significance set at *p*<0.05, SNP = single nucleotide polymorphism.

In the power calculations using the G*Power 3 program based on Cohen’s method [19], our sample size had > 80% power to detect a significant (α < 0.05) association for genotypes, alleles and haplotypes when an effect size index of 0.24 (corresponding to a “weak” gene effect) was used.

## Discussion

The presence of a complete ENS is required for a normal functioning intestinal tract throughout its entire length. The mammalian ENS is derived from a small pool of progenitor cells known as the enteric neural crest cells (ENCCs) [21]. It is indicated that normal ENS formation depends on an appropriate balance between ENCCs proliferation, differentiation and migration during ENS development, which is also based on the coordination of different signaling pathways, and defects in any of the pathway components might lead to Hirschsprung disease [22-24].

The Hedgehog (Hh) signaling pathway is fundamental for numerous processes during embryonic development, and is also implicated in mediating proliferation and differentiation of ENCCs [13,25,26]. Extracellular Hh proteins bind to the receptor PTCH1 (a protein of 1447 amino-acid residues containing 12 transmembrane domains and two large extracellular loops) and restrain PTCH1-mediated inhibition of downstream signaling by the transmembrane protein smoothened (SMO) [27]. Releasing SMO activity further activates the downstream cascade by promoting the formation of the activator form of the GLI-Kruppel family member GLI1 family of zinc-finger transcription factor [13]. Thus, aberrant activation or function of PTCH1 is supposed to induce the improper activation of the Hh signaling pathway [12]. Ramalho-Santos and colleagues [28] suggested appropriate Hh function was necessary for the formation of sufficient enteric ganglia in the intestinal tract, whereas excessive activation of the Hh pathway in ENCCs can cause progenitor progression. In addition, mice lacking either the Sonic Hh- or Indian Hh-secreted proteins presented partial intestinal aganglionosis, accompanied by megacolon or ectopic ganglia formation [28].

In this study, we present the first indication that common genetic variants in the *PTCH1* gene might confer altered susceptibility to Hirschsprung disease in the Han Chinese population and provide further support for the assumption that *PTCH1* might be involved in the etiology of HSCR. The genetic analysis was performed using the Sequenom MassARRAY technology (Sequenom, San Diego, CA), and altogether 8 genetic polymorphisms, including two SNPs previously investigated by Ngan et al. [13] and six other SNPs, were genotyped in the 104 subjects with HSCR and the 151 normal controls. At two of the eight markers (rs357565 and rs2236405), there were statistically significant discrepancies of allele or genotype frequencies between HSCR and control groups. In particular, we found the C allele of rs357565 and the A allele and AA genotype of rs2236405 were more frequent in the HSCR subjects compared to normal controls (Table 2), implying that all might be the risk factors for Hirschsprung disease.

We further carried out haplotype analysis in the genetic markers with strong LD (*D*’ > 0.7) since haplotypes constructed from closely located SNPs will typically increase the statistical power for association with the disease. Our data presented some significant global associations with HSCR and the most significant window spanned seven markers, giving a global *P* = 0.0036 (Table 5). Moreover, the most significant haplotype A-C-C-G-A-C-G-C (rs357552-rs357565-rs28485160-rs357564-rs2236405-rs28701981-rs2236407-rs10512248, *P* = 0.002, OR 5.52, 95% CI 1.66-18.35) was five times as common in the HSCR group (6.4%) as in the control group (1.3%), indicating it might be a risk haplotype for HSCR, whereas the haplotype A-A (rs357552-rs357565) might be a protective factor against Hirschsprung disease since a lower frequency observed in the HSCR subjects (12.2%) compared to the controls (22.8%) (*P* = 0.003, OR 0.47, 95% CI 0.29-0.78).

Out of the two positive markers we found in the present study, rs2236405 is a missense variant located in the third exon of *PTCH1* (Figure 1), and this SNP is an A>T transversion, changing a threonine residue to serine residue. The amino acid substitutions caused by missense variants are more likely to have serious consequences for the function or structural stability of the relevant protein [29,30]. Moreover, the other positive SNP, rs357565, is located in the untranslated region of *PTCH1*, and as non-coding regulatory variant rs357565 might also be functionally important, probably by affecting gene regulation and expression [29]. Ngan et al. [13] recruited canonical correlation analysis (CCA) to assess epistasis on data sets generated from a genome-wide association study on HSCR, and suggested that specific genotype constellations of *PTCH1* and *DLL3* (delta-like 3, a Notch ligand) SNPs conferred higher risk to HSCR. Rs357552 and rs10512248 included in the genotype constellations were also investigated in our study, and yet no positive results were observed at these two markers. However, since rs357565 is in strong LD with rs357552 (*D*’ = 0.96) (Table 3) and the correlated major alleles of both SNPs were implicated with a similar phenotype, we speculated that our findings might be regarded as an indirect support for the results observed by Ngan et al. [13].

In conclusion, our results provide a first indication that common genetic variants within *PTCH1* might confer altered risk to Hirschsprung disease in the Han Chinese population, further supporting *PTCH1* as a potential susceptibility gene for HSCR. If our findings can serve as a reference point for further replication studies in other ethnic groups and the mechanism can be verified in functional analyses, this could motivate genetic polymorphisms of *PTCH1* as molecular markers to reach early diagnosis of clinical manifestations.
